# Comparison of FDG PET/CT and Bone Marrow Biopsy Results in Patients with Diffuse Large B Cell Lymphoma with Subgroup Analysis of PET Radiomics

**DOI:** 10.3390/diagnostics12010222

**Published:** 2022-01-17

**Authors:** Eun Ji Han, Joo Hyun O, Hyukjin Yoon, Seunggyun Ha, Ie Ryung Yoo, Jae Won Min, Joon-Il Choi, Byung-Ock Choi, Gyeongsin Park, Han Hee Lee, Young-Woo Jeon, Gi-June Min, Seok-Goo Cho

**Affiliations:** 1 Division of Nuclear Medicine, Department of Radiology, Yeouido St. Mary’s Hospital, College of Medicine, The Catholic University of Korea, Seoul 06591, Korea; iwao@catholic.ac.kr; 2 Division of Nuclear Medicine, Department of Radiology, Seoul St. Mary’s Hospital, College of Medicine, The Catholic University of Korea, Seoul 06591, Korea; seunggyun.ha@gmail.com (S.H.); iryoo@catholic.ac.kr (I.R.Y.); thfus89@naver.com (J.W.M.); 3 Division of Nuclear Medicine, Department of Radiology, St. Vincent’s Hospital, College of Medicine, The Catholic University of Korea, Seoul 06591, Korea; atznawa@gmail.com; 4 Department of Radiology, Seoul St. Mary’s Hospital, College of Medicine, The Catholic University of Korea, Seoul 06591, Korea; dumkycji@gmail.com; 5 Department of Radiation Oncology, Seoul St. Mary’s Hospital, College of Medicine, The Catholic University of Korea, Seoul 06591, Korea; choibo67@catholic.ac.kr; 6 Department of Hospital Pathology, Seoul St. Mary’s Hospital, College of Medicine, The Catholic University of Korea, Seoul 06591, Korea; gspark@catholic.ac.kr; 7 Department of Gastroenterology, Yeouido St. Mary’s Hospital, College of Medicine, The Catholic University of Korea, Seoul 06591, Korea; hanyee99@hanmail.net; 8 Department of Hematology, Yeouido St. Mary’s Hospital, College of Medicine, The Catholic University of Korea, Seoul 06591, Korea; native47@catholic.ac.kr; 9 Department of Hematology, Seoul St. Mary’s Hospital, College of Medicine, The Catholic University of Korea, Seoul 06591, Korea; beichest@catholic.ac.kr (G.-J.M.); chosg@catholic.ac.kr (S.-G.C.)

**Keywords:** diffuse large B cell lymphoma, FDG, PET/CT, radiomics

## Abstract

Whether FDG PET/CT can replace bone marrow biopsy (BMBx) is undecided in patients with diffuse large B cell lymphoma (DLBCL). We compared the visual PET findings and PET radiomic features, with BMBx results. A total of 328 patients were included; 269 (82%) were PET-negative and 59 (18%) were PET-positive for bone lesions on visual assessment. A fair degree of agreement was present between PET and BMBx findings (ĸ = 0.362, *p* < 0.001). Bone involvement on PET/CT lead to stage IV in 12 patients, despite no other evidence of extranodal lesion. Of 35 discordant PET-positive and BMBx-negative cases, 22 (63%) had discrete bone uptake on PET/CT. A total of 144 patients were eligible for radiomic analysis, and two grey-level zone-length matrix derived parameters obtained from the iliac crests showed a trend for higher values in the BMBx-positive group compared to the BMBx-negative group (mean 436.6 ± 449.0 versus 227.2 ± 137.8, unadjusted *p* = 0.037 for high grey-level zone emphasis; mean 308.8 ± 394.4 versus 135.7 ± 97.2, unadjusted *p* = 0.048 for short-zone high grey-level emphasis), but statistical significance was not found after multiple comparison correction. Visual FDG PET/CT assessment and BMBx results were discordant in 17% of patients with newly diagnosed DLBCL, and the two tests are complementary in the evaluation of bone involvement.

## 1. Introduction

Diffuse large B cell lymphoma (DLBCL) is the most common aggressive lymphoma worldwide, accounting for approximately 30% of all adult non-Hodgkin’s lymphoma (NHL) cases. DLBCL is a biologically and clinically heterogeneous disease. Although rituximab plus cyclophosphamide, hydroxydaunomycin, vincristine, and prednisone (R-CHOP) is the standard first-line chemotherapy for patients with DLBCL, over one-third of patients show treatment failure after R-CHOP [1,2]. The management of lymphoma is evolving, and novel drugs with potential to change the therapeutic landscape for DLBCL are reported [3]. The most robust prognostic tool for DLBCL is the international prognostic index (IPI): age > 60, Eastern Cooperative Oncology Group (ECOG) performance status ≥2, Ann Arbor stage III or IV, elevated serum lactate dehydrogenase (LDH), and number of involved extranodal sites ≥2 are poor prognostic factors. The revised IPI is reported to represent high-risk patients receiving R-CHOP treatment for DLBCL [4]. Bone marrow (BM) is an extranodal site and leads to stage IV with higher IPI score and poor outcome. BM involvement has traditionally been diagnosed from biopsy obtained from the posterior iliac crest, considered the gold standard, and BM involvement is seen in up to 25% of DLBCL cases [5].

F-18-fluoro-2-deoxyglucose (FDG) positron emission tomography (PET)/computed tomography (CT) is used widely for evaluation and management of lymphoma. FDG PET/CT has high diagnostic accuracy for most aggressive types of lymphoma, and it is recommended over conventional imaging studies such as CT or magnetic resonance imaging (MRI) in many clinical scenarios including for DLBCL [6,7]. NCCN guideline suggests that when definite bone disease is present in the FDG PET/CT, BM biopsy (BMBx) is not necessary [8]. However, no clear criterion exists for diagnosing bone involvement on FDG PET/CT, and clinically FDG PET/CT has not fully replaced BMBx in the initial staging workup of patients with DLBCL.

Radiomics is a recently highlighted method of quantitatively analyzing images using an automated high-throughput extraction of large number of textural features from medical images. FDG PET radiomics has been studied to assess intratumoral heterogeneity and for prediction of treatment response and outcome in an oncologic context [9,10,11]. Recently, in hematologic application, FDG PET radiomics was utilized for differential diagnosis of primary lymphoma and other solid tumors [12]. Studies have looked into applying FDG PET radiomics of the BM to diagnose or predict the clinical outcome in patients with leukemia and multiple myeloma [13,14,15,16].

We aimed to compare the FDG PET/CT findings and BMBx results and explore multiple PET radiomic features from the posterior iliac crests and proximal femurs in patients with newly diagnosed DLBCL.

## 2. Materials and Methods

### 2.1. Patients

We retrospectively reviewed clinical records of patients with histologically confirmed DLBCL between January 2014 and December 2020 and a total of 338 consecutive patients had pre-treatment FDG PET/CT scans available. Of these, 10 patients who didn’t have BMBx results available for review were excluded from this study. Clinicopathologic variables such as age, sex, ECOG performance status, Ann Arbor stage, LDH titer, extranodal involvement, BMBx result, and IPI score at diagnosis were obtained from the medical records.

This study was performed in accordance with the approved guidelines of our hospital’s institutional review board. The ethical committee of our institution waived the need for patient consent for this retrospective review of imaging studies and clinical data.

### 2.2. FDG PET/CT Imaging Protocol

All patients fasted for at least 6 h. FDG (222–555 MBq) was injected intravenously and scanning began approximately 60 min later. No intravenous contrast agent was administered. Studies were acquired on integrated PET/CT scanners, Biograph Truepoint (Siemens Medical Solutions, Knoxville, TN, USA) or Discovery 710 (GE Healthcare, Milwaukee, WI, USA). All patients were in a supine position. CT began at the vertex and progressed to the upper thigh or toes using a standard protocol: 120 kV, 50 mA, 5 mm slice thickness (Biograph Truepoint); 120 kVp, variable mAs adjusted by topographic image, 2.5 mm slice thickness (Discovery 710). PET followed immediately over the same body region. Acquisition time was 2–3 min per bed position. The CT data were used for attenuation correction, and PET images were reconstructed using standard ordered-subset expectation maximization.

### 2.3. FDG PET/CT Image Analysis

All FDG PET/CT images were centrally reviewed using the same workstation (Mirada XD3; Mirada Medical, Oxford, UK). Two experienced nuclear medicine physicians, blinded to BMBx results and other clinical parameters, visually assessed the FDG PET/CT for bone involvement in each patient. If there was disagreement on visual assessment between two readers, consensus was made with a third nuclear medicine physician in presence. The readers visually assessed the bone status on FDG PET as positive or negative. PET-positive was defined as intense discrete uptake, either single or multiple in the bone(s), or diffusely increased FDG activity with intensity visually greater than the patient’s liver. PET-negative was defined as no or mild diffuse uptake in the bone(s) with intensity similar to or less than the liver [17].

### 2.4. Extraction of PET Radiomic Features

**Figure 1:**
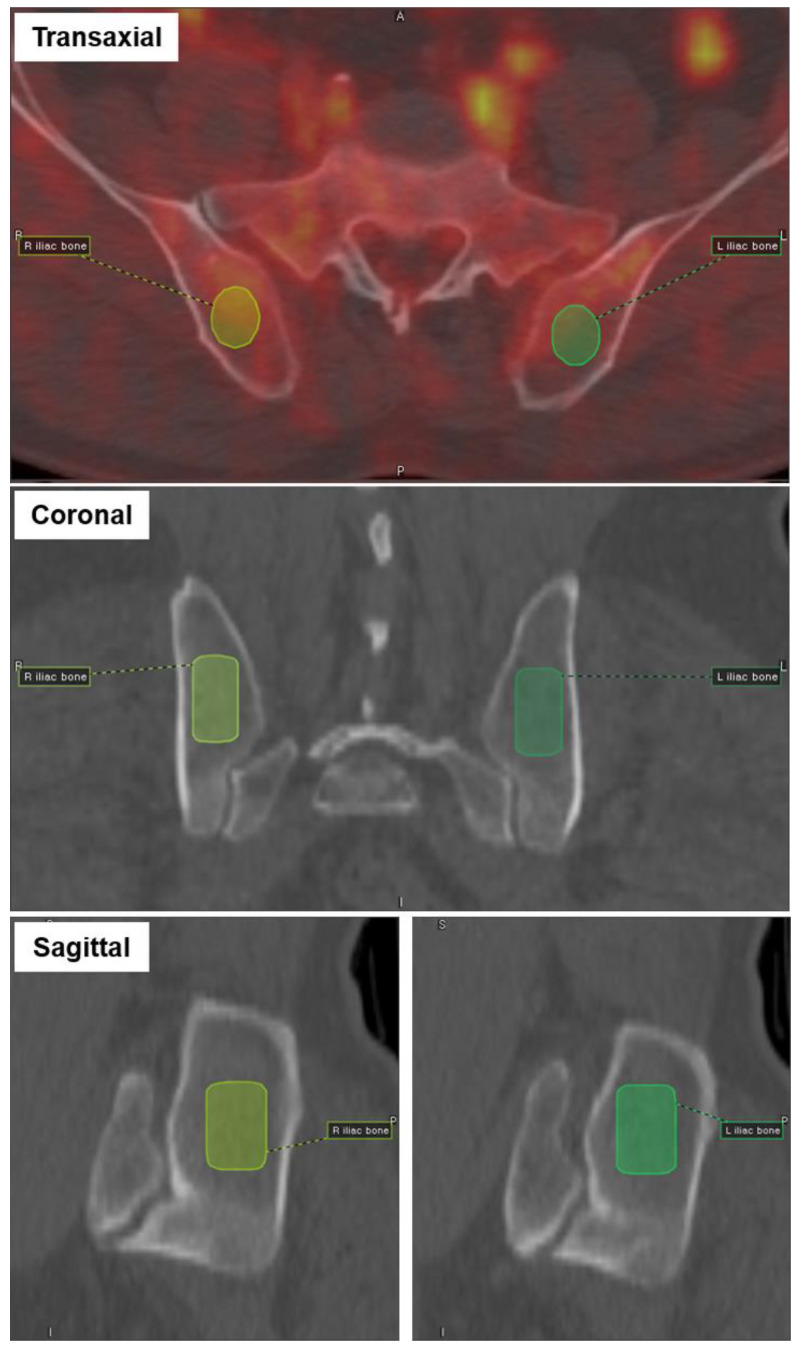
Cylinder volume of interests (VOIs) were placed on bilateral posterior iliac bones, and the CT images were reviewed to ensure the cortex of the bone was not included. The process was repeated to draw VOIs in bilateral femurs.

For the extraction of radiomic features from the BM on PET images, a dedicated open-source software (LIFEx version 6.38, www.lifexsoft.org (accessed on 14 September 2021) was used [18]. Cylinder volume of interests (VOIs) encompassing bilateral posterior iliac crests (3.5–4.0 cm^3^) and the proximal femurs (15–20 cm^3^) were manually drawn. The VOIs were inspected in the bone setting CT images to ensure the cortical bone was not included (Figure 1). The iliac bone was selected as it is the most common site for BMBx. The proximal femur was additionally selected to represent the BM activity of the long bones. The bone areas with the presence of prosthesis were excluded from the analysis to avoid PET attenuation correction artifacts.

For each patient, a total of 26 textural parameters were extracted from the four VOIs of bilateral posterior iliac crests and proximal femurs on PET images: two conventional imaging parameters (mean and maximum standardized uptake value [SUVmean and SUVmax]), four first-order textural features, six second-order textural features, and fourteen higher-order textural features. Four first-order textural features were extracted from intensity frequency histogram (skewness, kurtosis, entropy, and energy). Higher-order textural features included six grey-level co-occurrence matrix (GLCM) features, three neighborhood grey-level different matrix features (NGLDM), and eleven grey-level zone-length matrix (GLZLM) features. Grey-level run-length matrix (GLRLM) was not used to extract radiomic features because of its similarity to GLZLM (Table S1) [9,19,20].

### 2.5. Statistical Analysis

Categorical variables were expressed as an absolute number and percentage of the cases and continuous variables were expressed as mean ± standard deviation (SD) and range. The degree of agreement between BMBx and visual PET assessment was assessed using Cohen’s ĸ. A ĸ value of 0.0–0.2 was considered to represent slight agreement; 0.21–0.4, fair; 0.41–0.6, moderate; 0.61–0.8, substantial; and 0.81–1.0, almost perfect. Student’s *t*-test was used for comparison of textural parameters and false-discovery rate (FDR) control was applied for multiple comparison correction in radiomic analysis. All statistical analyses were performed using SPSS version 26.0 (IBM Corp., Armonk, NY, USA). Differences were considered statistically significant when the *p* value was less than 0.05.

## 3. Results

### 3.1. Patient Characteristics

A total of 328 patients (183 males, 145 females; mean age 59 ± 15 years) with pathologically confirmed DLBCL were included in this study. At baseline, 157 patients (47%) had advanced stage and BM involvement was confirmed by biopsy of posterior iliac crest in 45 (14%). MYC and BCL2 and/or BCL6 rearrangements (double/triple hits) were present in 17 patients (5%). The general characteristics of patients are in Table 1.

### 3.2. Visual Analysis of FDG PET/CT Images

On visual PET assessment, 269 patients (82%) showed bone with no or mild, diffuse pattern of FDG uptake with intensity similar to or less than the liver; 22 (7%) showed diffusely increased FDG uptake in the bones with intensity greater than the liver; and 37 (11%) showed discrete focal FDG uptake in the bone(s).

**Figure 2:**
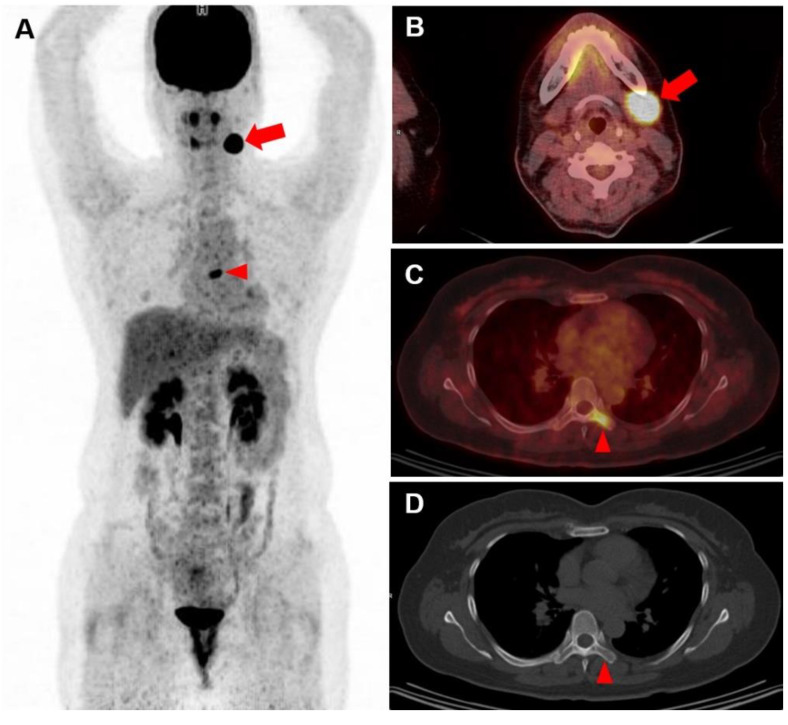
Baseline FDG PET/CT scan of a 48-year-old female patient with DLBCL confirmed by biopsy of left submandibular lymph node. Maximum intensity projection PET (**A**) and transaxial fusion PET/CT (**B**) images showed intense localized FDG uptake in left submandibular area (arrows; SUVmax 23.5). In addition, focal FDG uptake was noted in left transverse process and lamina of T6 vertebra (**C**; arrowhead; SUVmax 6.3), and considered as PET-positive on visual analysis. No abnormal lesion was detected on the corresponding bone setting CT image (**D**; arrowhead). However, she had BMBx-negative result.

Concordance analysis showed a fair agreement between visual assessment of FDG PET and BMBx in this study population (Cohen’s ĸ = 0.362, *p* < 0.001) with absolute agreement consistency of 83% (272 of 328) (Table 2). The number of discordant cases for FDG PET and BMBx tended to be higher in older patients (Table S2). In 12 patients (4%), bone involvement on FDG PET/CT led to clinical stage IV diagnosis, despite BMBx-negative results and no other imaging or clinical sign of extranodal involvement (otherwise stage III or below). Of 35 discordant cases with PET-positive and BMBx-negative findings, 22 (63%) had discrete focal bone uptake on FDG PET/CT images: 18 had one to five focal uptake sites (Figure 2), and other four had multifocal FDG uptakes throughout the axial and appendicular skeletons. The patients with double/triple hits had higher rates of discordant cases (5 of 17, 29%), compared to the rest of the study population (51 of 311, 16%).

### 3.3. Radiomic Analysis of FDG PET/CT Images

Of 222 patients with FDG PET/CT studies performed in the same system under the same acquisition and reconstruction conditions, 62 who underwent BMBx before PET/CT study and 16 with prosthesis causing attenuation artifacts were excluded from the radiomic analysis. A total of 144 patients were eligible for texture analysis, of which BMBx was positive in 23 patients (16%).

There was no significant difference between BMBx-positive and BMBx-negative patients in first- and second-order textural features extracted from iliac crests, including the SUVmean (1.7 ± 0.8 versus 1.3 ± 0.4), SUVmax (2.7 ± 1.6 versus 2.0 ± 0.7), skewness (0.3 ± 0.4 versus 0.4 ± 0.3), and homogeneity (0.4 ± 0.1 versus 0.4 ± 0.1) (all *p* > 0.05). Two GLZLM-derived indices, known as high grey-level zone emphasis (HGZE) and short-zone high grey-level emphasis (SZHGE), obtained from iliac crests showed a trend of higher values in BMBx-positive patients compared to BMBx-negative patients, but no statistical significance was found after multiple comparison correction (mean 436.6 ± 449.0 versus 227.2 ± 137.8, unadjusted *p* = 0.037 for HGZE; mean 308.8 ± 394.4 vs. 135.7 ± 97.2, unadjusted *p* = 0.048 for SZHGE; all FDR-adjusted *p* > 0.05). There was no significant difference between BMBx-positive and BMBx-negative patients for all the textural features extracted from the femurs.

## 4. Discussion

Routine clinical staging workup in patients with newly diagnosed lymphoma includes the evaluation of BM involvement. BMBx from iliac bone was reported to be positive in 10% to 25% of patients with newly diagnosed DLBCL and the result of this study is consistent with this range (14%; 45 of 328) [5,21]. In this study, visual assessment considered FDG PET to be positive for bone involvement in 18% (59 of 328) of the cases. Discordant rate between BMBx and FDG PET/CT was 17% (56 of 328), agreeing with previous studies that have shown that the agreement between bone FDG uptake and BMBx results are not very high [21,22]. Though there have been findings that FDG PET/CT is more sensitive than BMBx in patients with DLBLC, and recent recommendation states BMBx may no longer be indicated for the routine staging of Hodgkin’s lymphoma (HL) and most DLBCL [23,24,25], FDG PET/CT has not replaced BMBx in the clinical practice of many hematology centers. The value of BMBx in DLBCL goes well beyond the sole detection of bone involvement. BMBx could identify a low-volume marrow infiltrate or underlying indolent lymphoma [21,26], and provide information on the presence of myelodysplastic features [27]. Such findings could guide the therapeutic plan and management of toxicities. Of the 56 discordant cases in our study, 21 cases had BMBx-positive results while the FDG PET was negative for any bone lesion. Low-volume (10–20%) diffuse BM involvement on pathologic examination was reported to cause false negative PET findings [28,29]. Conversely, 35 other discordant cases had BMBx-negative but PET-positive findings. Among these cases, there were 22 cases with discrete focal FDG uptake in the bone on FDG PET/CT images, and clinically considered to be BM/bone positive. Eighteen of these 22 patients showed focal FDG uptakes in five or fewer bone sites, and another four patients had FDG uptakes in 10 or more sites. Biopsy from iliac bone alone cannot fully represent the heterogeneous biological behavior of multiple bones [30]. Limitations of BMBx include site-dependence and discordance in morphology between lymphomatous cells in extramedullary sites and those cells in the BM. In addition, histological data may report a different pathologic non-lymphoma-related malignancy or disorder. The invasive nature, discomfort or pain for the patients, and dependence on the clinical expertise of the biopsy performer are also limitations of BMBx [21,31]. In one previous study of 140 patients with DLBCL, the group with BMBx-positive and bone PET-positive findings had worse progression-free survival (PFS) than the group with BMBx-positive but PET-negative findings (median PFS 7.4 versus 13.9 months, *p* = 0.04). BMBx-negative and PET-positive patients had similar outcomes compared to BMBx-positive and PET-negative patients [32]. The results suggest complementary roles of biopsy and FDG PET/CT in assessment of the BM/bones in DLBCL.

**Figure 3:**
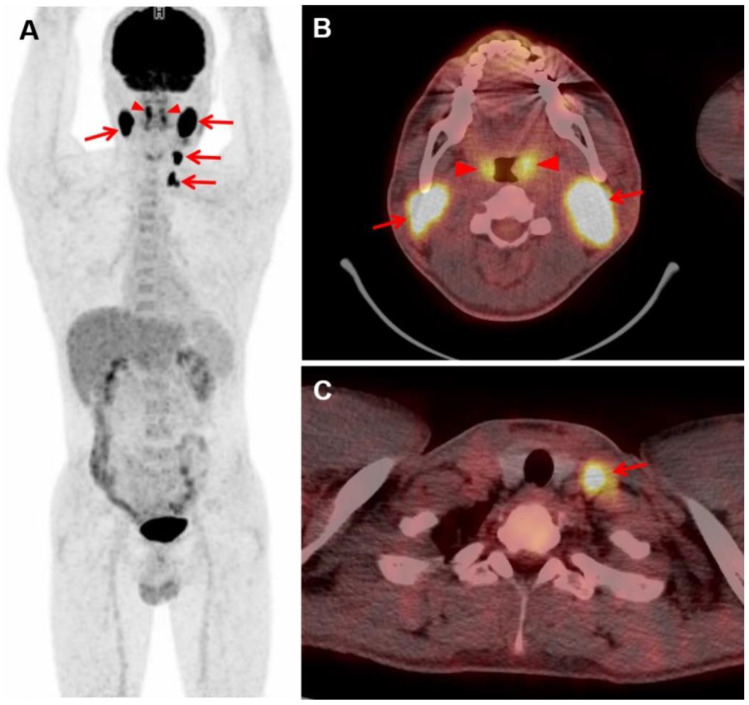
Baseline FDG PET/CT scan of a 36-year-old male patient with DLBCL confirmed by biopsy of the left tonsil. In maximum intensity projection PET (**A**) and transaxial fusion PET/CT (**B**,**C**) images, bilateral tonsils showed increased FDG uptake (arrowheads) and enlarged bilateral cervical lymph nodes showed intensely increased FDG uptake (arrows; SUVmax 23.2 in right cervical level II). The bone FDG uptake had intensity less than the liver and was considered BM PET-negative on visual analysis. However, he had BMBx-positive result. In radiomic analysis, two GLZLM-derived indices obtained from iliac crests were relatively high (HGZE 359.3 and SZHGE 229.0).

What to consider abnormal FDG uptake of the BM and bones and the clinical significance of diffuse FDG uptake of the BM seen on the baseline FDG PET/CT scan are issues without clear consensus currently [33,34]. Many previous studies considered discrete focal bone uptake exceeding hepatic uptake in intensity as indicative of BM involvement of lymphoma, and we also considered the liver intensity as reference point. However, it is well known that inflammatory or infectious conditions and abnormality of the hematologic parameters are associated with BM hyperplasia that can manifest as increased FDG uptake on PET/CT [35,36]. A previous study of 512 patients with DLBCL had the FDG uptake in the BM assessed, and PET-positive was defined as focal/diffuse BM activity ≥ normal liver. In subgroup analysis with 59 BMBx-positive patients, leukopenia and pancytopenia were significantly more frequent in those with increased FDG uptake in the bones. Same tendency was noted for anemia and thrombocytopenia, and the authors suggested applying higher threshold for associating clinical significance with diffusely increased BM FDG uptake [37]. A multi-center study by Chen-Liang et al. [38] looked into 372 patients with high-grade NHL and HL and proposed the following management scheme: (a) focal FDG uptake (+) and stage IV, do not undergo BMBx; (b) focal/diffuse FDG uptake (+) and SUVmax < 4, undergo BMBx; and (c) FDG uptake (-), then undergo BMBx. From our study population, there were three patients with diffuse FDG uptake with SUVmax > 4, a category not specified in the above-mentioned scheme, and two of the three patients had BMBx-positive results. Diffuse FDG uptake with SUVmax < 4 was seen in 18 patients, and six had BMBx-positive results in our study. For patients with negative bone FDG uptake, the BMBx-positive yield was 7.8% (21 of 269) (Figure 3).

In addition to visual assessment of FDG PET/CT, analysis of radiomic features extracted from FDG PET images of iliac bones, the sites corresponding to BMBx, was performed and two GLZLM-derived indices, known as HGZE and SZHGE, showed significantly higher values in patients with BMBx-positive results compared to those with BMBx-negative results in this study. These findings suggest the possibility that PET radiomic features augmenting the regional high intensity level may potentially be informative of BM characteristics. However, this result was not statistically significant after multiple comparison correction, and our study could not provide a conclusion with satisfactory statistical validation. Further study refined for HGZE and SZHGE as PET biomarkers of BM involvement and subsequent validation are required. Additional evaluation is also required to see if these radiomic features can help select patients who will benefit from BMBx in the setting of negative FDG PET on visual assessment. There are few studies on PET radiomics for evaluation of BM involvement in patients with lymphoma. Mayerhoefer et al. [39] looked into 97 patients with mantle cell lymphoma for prediction of BM involvement using PET radiomics. When the cut-off percentage of cellular BM involvement was set low to 5%, PET radiomic feature (GLCM) showed better classification performance than SUVs alone. Kenawy et al. [40] showed in a study with 44 patients with lymphoma that PET radiomic features and visual assessment are synergistic for characterizing BM involvement. Aide et al. [41] limited the study to 82 patients with DLBCL, and in multivariate analysis, the only independent predictor of PFS was skewness and the only independent predictor of overall survival (OS) was the IPI score. In the study by Aide et al., 18% patients with both BMBx-negative results and PET-negative findings were falsely considered positive for BM involvement when skewness was applied, but these patients had poorer PFS and OS than patients with all negative findings on BMBx, visual PET assessment, and skewness. Previous studies and our findings suggest that PET radiomics could contribute to better depicting the BM involvement in addition to biopsy and visual assessment, but further studies are necessary.

The main limitation of this study was its retrospective design and relatively small number of cases included in radiomic analysis due to different PET/CT scanners used in the clinic. Further studies with larger sample sizes and clinical follow-up could result in more conclusive radiomic analysis.

## 5. Conclusions

Visual PET assessment and BMBx results were discordant for bone involvement in 17% patients (56 of 328) with newly diagnosed DLBCL. FDG PET/CT and BMBx are complementary in the evaluation of BM involvement of lymphoma.

## Figures and Tables

**Table 1:** General characteristics of 328 patients.

Variables		Number (%)
Age	Mean ± SD (range)	59 ± 15 years (21–94)
Sex	Male	183 (56%)
	Female	145 (44%)
ECOG PS	0–1	283 (86%)
	2–4	45 (14%)
Ann Arbor stage	I	77 (23%)
	II	94 (29%)
	III	44 (13%)
	IV	113 (34%)
LDH titer ^1^	Normal	150 (46%)
	Elevated	177 (54%)
Extranodal involvement	0–1	199 (61%)
	>1	129 (39%)
Bone marrow biopsy	Negative	283 (86%)
	Positive	45 (14%)
IPI score	Low or low-intermediate risk (0–2)	194 (59%)
	High-intermediate risk (3)	70 (21%)
	High risk (4–5)	64 (20%)

ECOG PS: Eastern Cooperative Oncology Group performance status; LDH: lactate dehydrogenase; IPI: international prognostic index; SD: standard deviation. ^1^ Unavailable in one patient.

**Table 2:** Visual bone assessment on FDG PET and BMBx results.

	BM PET-Negative	BM PET-Positive	Total
**BMBx-negative**	248 (76%)	35 (11%)	283 (86%)
**BMBx-positive**	21 (6%)	24 (7%)	45 (14%)
**Total**	269 (82%)	59 (18%)	328 (100%)

## Data Availability

The data from this study are available from the corresponding author upon reasonable request.

## Supplementary Materials

The following are available online at https://www.mdpi.com/article/10.3390/diagnostics12010222/s1, Table S1: The extracted radiomic features; Table S2: Number of discordant cases by age.
