# Supplementary material for: Comparison of FDG PET/CT and Bone Marrow Biopsy Results in Patients with Diffuse Large B Cell Lymphoma with Subgroup Analysis of PET Radiomics
Source: Diagnostics (Basel). 2022 Jan 17;12(1):222. doi: 10.3390/diagnostics12010222 (PMC8774933; doi:10.3390/diagnostics12010222)
Supplement: Supplementary file 1 [file diagnostics-12-00222-s001.zip › diagnostics-1500830-supplementary.pdf]

Table S1. The extracted radiomic features.

Conventional (n=2)	Mean standardized uptake value<br>Maximum standardized uptake value
Histogram (n=4)	Skewness<br>Kurtosis<br>Entropy<br>Energy
GLCM (grey-level co-occurrence matrix) (n=6)	Homogeneity<br>Energy<br>Contrast<br>Correlation<br>Entropy<br>Dissimilarity
NGLDM (neighborhood grey-level different matrix) (n=3)	Coarseness<br>Contrast<br>Busyness
GLZLM (grey-level zone-length matrix) (n=11)	SZE (short-zone emphasis)<br>LZE (long-zone emphasis)<br>LGZE (low grey-level zone emphasis)<br>HGZE (high grey-level zone emphasis)<br>SZLGE (short-zone low grey-level emphasis)<br>SZHGE (short-zone high grey-level emphasis)<br>LZLGE (long-zone low grey-level emphasis)<br>LZHGE (long-zone high grey-level emphasis)<br>GLNU (grey-level non-uniformity for zone)<br>ZLNU (zone length non-uniformity)<br>ZP (zone percentage)

Table S2. Number of discordant cases by age.

Age (y)	Total	Discordant case
21-30	15	2 (13%)
31-40	28	3 (11%)
41-50	45	7 (16%)
51-60	81	12 (15%)
61-70	78	11 (14%)
71-80	71	17 (24%)
81-90	9	3 (33%)
91-	1	1 (100%)
Total	328	56
